# Endophytic Consortium With Diverse Gene-Regulating Capabilities of Benzylisoquinoline Alkaloids Biosynthetic Pathway Can Enhance Endogenous Morphine Biosynthesis in *Papaver somniferum*

**DOI:** 10.3389/fmicb.2019.00925

**Published:** 2019-04-30

**Authors:** Tania Ray, Shiv S. Pandey, Alok Pandey, Madhumita Srivastava, Karuna Shanker, Alok Kalra

**Affiliations:** ^1^Microbial Technology Department, CSIR-Central Institute of Medicinal and Aromatic Plants, Lucknow, India; ^2^Biotechnology Division, CSIR-Central Institute of Medicinal and Aromatic Plants, Lucknow, India; ^3^Analytical Chemistry Department, CSIR-Central Institute of Medicinal and Aromatic Plants, Lucknow, India

**Keywords:** endophytes, consortium, benzylisoquinoline alkaloids, morphine, *Papaver somniferum*, opium

## Abstract

Secondary metabolite biosynthesis in medicinal plants is multi-step cascade known to be modulated by associated endophytes. While a single endophyte is not able to upregulate all biosynthetic steps, limiting maximum yield achievement. Therefore to compliment the deficient characteristics in an endophyte we tried consortium of endophytes to achieve maximum yield. Here, efforts were made to maximize the *in planta* morphine yield, using consortium of two endophytes; SM1B (*Acinetobacter* sp.) upregulating most of the genes of morphine biosynthesis except *T6ODM* and *CODM*, and SM3B (*Marmoricola* sp.) upregulating *T6ODM* and *CODM* in alkaloid-less *Papaver somniferum* cv. Sujata. Consortium-inoculation significantly increased morphine and thebaine content, and also increased the photosynthetic efficiency of poppy plants resulted in increased biomass, capsule weight, and seed yields compared to single-inoculation. The increment in morphine content was due to the modulation of metabolic-flow of key intermediates including reticuline and thebaine, via upregulating pertinent biosynthetic genes and enhanced expression of *COR*, key gene for morphine biosynthesis. This is the first report demonstrating the endophytic-consortium complimenting the functional deficiency of one endophyte by another for upregulating multiple genes of a metabolic pathway similar to transgenics (overexpressing multiple genes) for obtaining enhanced yield of pharmaceutically important metabolites.

## Introduction

Increasing populations have threatened food security due to the availability of limited land for cultivation. Moreover, changing environmental conditions are also causing a considerable reduction in the crop productivity. It is estimated that by the year 2050, the world population may reach nine billion. Therefore, to satisfy the needs of the growing population diverse approaches have been tried to increase the food production at the same or even higher rates. Developing transgenics has so far received foremost attention by introducing desired traits to produce the desired product and improving the productivity in normal as well as changing environmental conditions including cultivation under biotic and abiotic stresses. Plant yield and tolerance to different environmental stresses are polygenic traits and regulated by multiple genes (Bohnert et al., 2006). Therefore, integration/expression/genetic manipulation of more than one genes in a plant became the preferred choice for improvement of plant yield and performance under stressful environmental conditions. However, these practices have limitations related to production cost, social acceptability, and sustainability (Halpin, 2005). Therefore, besides developing transgenic plants, alternate sustainable approaches need to be explored for achieving maximum plant yield.

Endophytes have emerged as a promising candidate for sustainable agriculture. Endophytes are plant-associated microbes residing within the plant without harming or causing any disease symptoms. They promote plant growth, protect plants from environmental stresses and are the promising source of therapeutic secondary metabolites (Kuklinsky-Sobral et al., 2004; Strobel et al., 2004; Waller et al., 2005; Rodriguez et al., 2008; Staniek et al., 2008; Kusari et al., 2012; Quecine et al., 2012; Otieno et al., 2015; Egamberdieva et al., 2017). Involvement of endophytes in the modulation of secondary metabolite biosynthesis of the host plant has also been established (Pandey et al., 2016a,b, 2018). Bacterial endophyte interaction with *E. purpurea* enhances its secondary metabolites which contributes to therapeutic properties of this medicinal plant (Maggini et al., 2017). An *Artemisia annua* endophytic actinobacterium *Pseudonocardia* strain YIM 63111 has been able to induce artemisinin production by upregulating the genes of artemisinin biosynthetic pathway, i.e., cytochrome P450 monooxygenase and cytochrome P450 oxidoreductase (Li et al., 2012). Also role of microbial community present in the cortical parenchymatous tissue of *Vetiver* in essential oil biosynthesis has been reported (Del Giudice et al., 2008). Inoculation with bacterial endophytes increases biomass and in planta content of terpenoid indole alkaloids like vindoline in *Catharanthus roseus* (Tiwari et al., 2013). *Paenibacillus strain* has been found to strongly influence the plant metabolites of *in-vitro* grown poplar plants (Scherling et al., 2009). Interestingly, endophytes are also able to produce secondary metabolites analogous to their host plants, e.g., camptothecin and analogs (Shweta et al., 2010), taxol (Soliman et al., 2011), vincristine, and vinblastine (Kumar et al., 2013). Fungal endophytes as symbiotically modified organism (SMO) have also been used to improve grass yield, provide resistance against pests, weeds and herbivores by accumulation of alkaloids and antioxidants (Gundel et al., 2013, 2018). It has been demonstrated in our laboratory that endophytes are involved in the improvement of the content of vindoline (intermediate for the biosynthesis of anticancerous vinblastine and vincristine) in *Catharanthus roseus* (Pandey et al., 2016a), withanolides in *Withania somnifera* (Pandey et al., 2018), and benzylisoquinoline alkaloids (BIAs) in *Papaver somniferum* (Pandey et al., 2016b). Previous studies have also demonstrated the diverse role of endophytes in a host plant (Di Fiore and Del Gallo, 1995; Saikkonen et al., 1998; Singh et al., 2011;Hardoim et al., 2015; Pandey et al., 2016a,b, 2018). Every endophyte has a specific characteristic, up/down-regulating certain specific genes responsible for plant growth, yield, and modulation of secondary metabolite biosynthesis. Although reports signifying the role of a particular endophyte modulating a single functional characteristic like growth promotion or disease resistance, stress tolerance or enhance secondary metabolite production are available, but the information on the possibility of strengthening multiple functional characteristics through the use of consortium is scarce. Therefore, the use of consortium of endophytes having different functional characteristics should be explored to maximize the yields and desired products.

Here, we demonstrated the use of consortium of selected endophytes associated with the capsule of opium poppy plant which is the site for BIA biosynthesis to maximize the yield of pharmaceutically important metabolite morphine. Opium poppy is an important medicinal plant and sole source of therapeutically important BIAs such as morphine, codeine, thebaine, papaverine, noscapine, and sanguinarine. Morphine belongs to the group “opioids” which are key drugs for the alleviation of moderate-to-severe acute pain associated with cancer and other diseases or after any surgery or physical trauma (Rosenblum et al., 2008; Whittle et al., 2011).

Previously we have established that the opium poppy has potential endophytes which can enhance *in planta* BIA biosynthesis. We demonstrated that different endophytes are associated with different parts of poppy plants performing an important role in a tissue-specific manner. In general, endophytes related with leaf were involved in modulating photosynthetic efficiency and endophytes associated with the capsule were drawn in modulating the BIA-biosynthesis of the poppy plant. It was observed that endophytes SM1B and SM3B isolated from capsule of alkaloid rich Sampada were unable to enhance the BIA production in *P. somniferum* cv. Sampada plants. However, these capsule-associated endophytes were able to increase the production of BIAs in alkaloid-less cv. Sujata. The cv. Sujata was developed from alkaloid-rich cv. Sampada with the aim to convert a narcotic “opium poppy” (i.e., cv. Sampada) into non-narcotic seed poppy (i.e., cv. Sujata) using gamma rays and EMS (ethyl methane sulphonate) (Sharma et al., 1999). The cv. Sujata is considered to be having the potential for commercial cultivation of seeds due to high nutritive value of 24% protein, >58% oil with high unsaturated fatty acids makes it a safe and potential food crop having protein rich seeds (Sharma et al., 2002). Previously we have established that the individual inoculation with endophytes SM1B and SM3B increased biomass upto 44% in cv. Sujata. SM1B inoculation in cv. Sujata plants increased content of Morphine by 1044%, Papaverine by 349% and Noscapine by 936%, and SM3B inoculation increased Morphine by 37%, Papaverine by 66%, Noscapine by 72%, and Thebiane by 154% (Pandey et al., 2016b). It was observed that endophyte SM1B could upregulate most of the genes of BIA biosynthesis except thebaine 6-O-demethylase (*T6ODM*) and codeine O-demethylase (*CODM*) (Pandey et al., 2016b). However, we could also identify another endophyte SM3B that was able to upregulate *T6ODM* and *CODM* expression. We, therefore, expected that the consortium of SM1B and SM3B will lead to the overexpression of all BIA biosynthetic pathway genes simultaneously and will produce more morphine than single inoculation. Here, efforts were made to maximize morphine yield using the consortium of two capsule-associated endophytes SM1B (*Acinetobacter* sp.) and SM3B (*Marmoricola* sp.) in the poppy plant by upregulating different genes of BIA biosynthesis.

## Materials and Methods

### Plant Materials and Growth Conditions

Seeds of *Papaver somniferum* cv. Sujata was obtained from the National Gene Bank for Medicinal and Aromatic Plants at CSIR-CIMAP, Lucknow. Poppy plants were grown in pots filled with 3.5 kg of autoclaved soil and vermicompost mixture (2:1, v/v) having dimension (17 cm height × 22 cm top diameter × 12 cm bottom diameter and 3.7 l volume) and irrigated with sterile water with an ordinary photoperiod in a greenhouse under natural light intensity at 20°C ± 2°C.

### Antagonistic Activity of Endophytes

Antagonistic activity of both the selected endophytes was tested by *in vitro* antimicrobial assay. For this, the disc diffusion method of Bhunia et al. (1988) was followed with some modifications. SM1B and SM3B both were grown in nutrient broth for 24 h at 28°C. The final concentration of each bacterium was adjusted to 10^6^ CFU/ml. 100 μl 10^6^ CFU/ml cell suspension was spread on nutrient agar media and leave the plates to get dried on room temperature (Tajehmiri et al., 2014**)**. The sterile paper disc (6 mm) were placed over nutrient agar media plates, seeded with indicator strains. Hundred microliter of culture was added to the sterile paper disc and incubated at 28°C for 48 h. After incubation, antagonistic activity was observed around the paper disc. As there was no zone of inhibition around the paper disc and both the bacteria were growing with each other which confers that there is no antagonistic activity between the two bacterial strains and they synergistically performed their function.

### Endophyte Inoculation

For consortium development individual suspension (1 × 10^8^ CFU ml^−1^) of the two endophytes (SM1B and SM3B) was prepared in PBS and were mixed at a CFU of 1 × 10^8^ml^−1^ microbes each in 5 ml culture prepared in PBS. Endophyte-free seeds were treated by putting them in consortium suspension for 2 h and then used for sowing in pots. Pots were kept in the controlled condition in greenhouse. For each treatment the experiment was conducted in triplicates. Initially, there were 25 seeds sown in each pot, and when the seeds grown for 2 weeks. Only five healthy seedlings were kept in each pot. In order to boost up the soil with sufficient inoculum of endophyte, to ensure their presence in the soil. The pots were again inoculated with 10 ml pot^−1^ of the endophyte suspension (1 × 10^8^ CFU ml^−1^). To make certain the presence of adequate numbers of endophyte in the soil, pots were inoculated for a second time after 2 weeks of germination of seeds. PBS was used in place of endophyte suspension in case of the control plants (uninoculated). To confirm the presence of specific endophytes, re-isolation of endophyte has been done from various parts of poppy plants at 60 days stage. No endophytes were found to be present at any stage from the uninoculated endophyte-free control plants. SM1B and SM3B inoculated plants (individual inoculation) were grown simultaneously with the consortium-inoculated (SM1B and SM3B) plants.

### Colonization of Endophytes

Endophyte colonization was inspected in the capsule of poppy plants in triplicates after 90 days of growth. Tissues were surface sterilized and sterility check was performed as illustrated previously (Pandey et al., 2016b). Under sterile conditions, the surface sterilized tissues were thoroughly macerated, and serial dilutions were plated on nutrient agar with three replications each. The plates were then incubated at 28°C for 24–72 h and then the CFU were estimated. The obtained colonies were confirmed by the morphological characteristics followed by 16S rRNA sequencing.

### Photosynthetic Pigment Content, Photosynthetic Rate, Stomatal Conductance, Transpiration Rate

Photosynthetic pigment (Chlorophyll) content and photosynthetic efficiency of fully expanded leaves (third from the top) of 60 days poppy plants (*n* = 6) were measured. Chlorophyll extraction was performed in chilled methanol, and the content was estimated (Lichtenthaler and Buschmann, 2001). Photosynthetic efficiency (net CO_2_ assimilation, transpiration rate and stomatal conductance) was measured in the attached leaves (third from the top) using the Portable Photosynthesis System (CIRAS-3, PP System, United States) attached with the Chlorophyll fluorescence module (CFM-3). For photosynthesis measurement, CO_2_ concentration in leaf cuvette was maintained at ambient CO_2_ (400 ppm), temperature (25°C) and 400 μmol photons m^−2^ s^−1^ light.

### Biomass, Capsule Weight, and Seed Yield

Biomass of 60 days old poppy plant (*n* = 6) was measured by harvesting the entire shoots and dried at 70°C for 5 days, and their dry weights were measured. Capsule weight and seed yield were measured from 120 days mature poppy plants.

### Benzylisoquinoline Alkaloids

BIAs analysis was performed from the capsules of 120 days old poppy plants. Capsules were completely dried and ground to fine powder. Powdered samples (1 g) were extracted three times in 100% methanol (30 ml) with 20 min sonication at 42°C. Extract was filtered through Whatman Number 1 filter paper and then the filtrates were concentrated in Rotavapour, dried, and re-dissolved in methanol. BIAs (morphine, codeine, thebaine, papaverine, noscapine and reticuline) were analyzed by isocratic reverse-phase HPLC using phosphate buffer (0.1 M NaH_2_PO_4_.2H_2_O, pH 3.5, 77%, v/v) and acetonitrile (23%, v/v), sample injection volume of 20 μl, at flow rate of 1.0 ml min^−1^ on Phenomenex, Luna C_18_ column^^®^^, Waters (4.6 × 250 mm, 5 μm). The analysis was performed with Empower Pro software (Waters, United States). Photodiode array data acquisition was in the range of 200–400 nm and the quantitation was done at 240 nm. Peaks of analyzed alkaloids were identified by comparing their retention times with commercially available authentic standards. Noscapine and reticuline were purchased from Sigma-Aldrich, St. Louis, United States. Morphine, codeine, thebaine and papaverine were gifted from CSIR-National Botanical Research Institute, Lucknow. The data was an average of two independent quantifications repeated in three biological replicates (*n* = 6).

### qRT-PCR Analysis

Total RNA was isolated from the green capsule of un-inoculated endophyte-free control and endophytes-inoculated 90 days old poppy plants by using TRI-reagent (Sigma-Aldrich). RNA quantification was done using NanoDrop 1000 spectrophotometer (Thermo Fisher Scientific). Genomic-DNA contamination in the total RNA preparation was eliminated using RNase-free DNase I enzyme (Thermo Scientific). First-strand cDNA synthesis was performed with ∼5 μg of total RNA using the RevertAid First Strand cDNA Synthesis Kit with oligo(dT)_18_ primer (Thermo Scientific) following the manufacturer’s instructions. Previously described gene-specific primers were used for the analysis of relative quantification of 16 biosynthetic gene transcripts involved in BIA biosynthetic pathway (Pandey et al., 2016b). qRT-PCR was performed using SYBR-Green I detection on triplicate technical replicates of triplicate biological samples (*n = 3*) on an Applied Biosystems StepOnePlus^TM^ Real-Time PCR System as described previously (Pandey et al., 2016b). PCR mixture contains 1 μl of 10 times diluted cDNA synthesis reaction, 300 nM each forward and reverse primers and 5 μl Power SYBR Green PCR Master Mix (Applied Biosystems), in a 10 μl reaction volume. PCR conditions were maintained at 95°C for 10 min, followed by 40 cycles of denaturation at 95°C for 15 s each and annealing at 60°C for 1 min each. The intensities of fluorescent signal were recorded on an Applied Biosystems StepOnePlusTM Real Time PCR System and analysis were carried out using StepOne software (Applied Biosystems). Specificity of RT-qPCR was evaluated by using the dissociation method (Applied Biosystems) subjecting all amplicons to a melt-curve analysis. The threshold cycle (Ct) for each gene was normalized against the Ct for β-actin (EB740770) of opium poppy, which was used as the endogenous reference transcript. Further, Mean Ct values were calculated from technical triplicates, and the relative levels of transcripts of different genes. BIAs biosynthetic genes in endophyte-inoculated plants were compared with the calibrator (non-inoculated control plant) using the relative quantification 2^−ΔΔCt^ method and efficiency of PCR was also determined by the method of Livak and Schmittgen (2001) and Schmittgen and Livak (2008). For amplification serially diluted cDNA was used as template for different target genes and reference gene. To determine ΔCt (Ct target – Ct reference) the average Ct was calculated for both target gene and reference gene. A plot of the log cDNA dilution versus ΔCt was drawn, and the absolute value of the slope were found to be near zero for all the target genes that showed that the efficiencies of the target and reference genes were quite similar. Therefore, relative quantification 2^−ΔΔCt^ method was applied to analyze the data (Livak and Schmittgen, 2001).

### Statistical Analysis

Statistical analysis of data was carried out following ANOVA analysis of variance, suitable to completely randomized design (CRD), using SPSS (IBM, Chicago, IL, United States). Comparisons between means were carried out using Duncan’s multiple range tests at a significance level of *P* ≤ 0.05.

## Results

### Antagonistic Activity of Endophytes and Their Colonization in the Capsule of Poppy Plants

**FIGURE 1:**
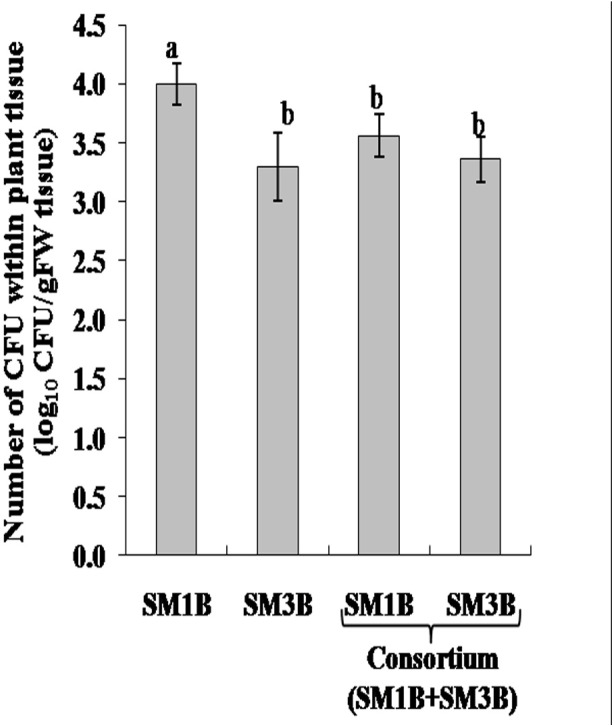
Colonization of endophytes in the capsule of *Papaver somniferum* plants. CFU- colony forming unit. Values are the mean ± SE of three replicates (*n* = 3). Different letters (a–b) indicate statistically significant differences between treatments (Duncan’s multiple range test *P* < 0.05).

Antagonistic activity of SM1B and SM3B was tested. It was observed that both endophytes grew well together and did not inhibit the growth of each other (Supplementary Figure S1). Colonization of SM1B and SM3B endophytes was determined in the capsule of poppy plant inoculated with SM1B and SM3B individually as well as a consortium of SM1B and SM3B. It was observed that both endophytes could successfully colonize the capsule of *P. somniferum* plant (Figure 1). Colonization of SM1B was reduced in the poppy plants inoculated with a consortium of SM1B and SM3B compared to individual inoculation of SM1B. However, the colonization of SM3B was found to be similar in both individual (SM3B), and consortium inoculated plants (Supplementary Table S1).

### Effect of a Consortium of Endophytes on Photosynthetic Efficiency of the Plant

Photosynthetic efficiency of plants was studied by measuring the chlorophyll content, net CO_2_ assimilation rate, stomatal conductance, and transpiration rate. Application of endophytic consortium improved the measured parameters. Inoculation with the consortium of SM1B and SM3B in *P. somniferum* plants increased chlorophyll content, net CO_2_ assimilation rate, stomatal conductance, and transpiration rate by 27.1, 25.8, 39.3, and 46.5%, respectively, compared to endophyte-free control plants (Table 1). Individual inoculation of SM1B could increase the chlorophyll (21.43%), net CO_2_ assimilation rate (15.63%), stomatal conductance (30.36%), and transpiration rate (21.81%), however, SM3B inoculation could only increase the chlorophyll content (17.14%).

**Table 1:** Effect of inoculation of consortium of endophytes on different physiological parameters of *Papaver somniferum* plant.

Physiological parameters	Control	SM1B	SM3B	Consortium (SM1B+SM3B)
Chlorophyll (mg gFW^−1^)	0.70 ± 0.03^b^	0.85 ± 0.06^ab^	0.82 ± 0.03^ab^	0.89 ± 0.04^a^
Net CO_2_ assimilation (μMole CO_2_ m^−2^s^−1^)	7.87 ± 0.16^c^	9.10 ± 0.12^b^	7.90 ± 0.24^c^	9.90 ± 0.11^a^
Stomatal conductance (mMole m^−2^s^−1^)	321.67 ± 2.86^c^	419.33 ± 1.22^b^	325.67 ± 7.35^c^	448.00 ± 6.12^a^
Transpiration rate (mMole m^−2^s^−1^)	3.53 ± 0.20^c^	4.30 ± 0.10^b^	3.56 ± 0.06^c^	5.17 ± 0.06^a^
Biomass (g Plant^−1^)	0.60 ± 0.04^c^	0.79 ± 0.03^b^	0.68 ± 0.02^c^	1.05 ± 0.01^a^
Capsule weight (g Plant^−1^)	1.22 ± 0.12^c^	1.59 ± 0.02^b^	1.18 ± 0.09^c^	2.03 ± 0.11^a^
Seed yield (g Plant^−1^)	0.52 ± 0.06^b^	0.66 ± 0.02^b^	0.57 ± 0.04^b^	0.93 ± 0.09^a^

*Different letters (a–c) indicate statistically significant differences between treatments (Duncan’s multiple range test P < 0.05). Data are means ± SD (n = 6).*

### Effect of a Consortium of Endophytes on Biomass, Capsule Weight, and Seed Yield

Application of consortium of endophytes significantly enhanced the biomass, capsule weight and seed yield of *P. somniferum* plants. Inoculation with a consortium of SM1B and SM3B increased the biomass of *P. somniferum* plant by 75% compared to endophyte-free control plants (Table 1). Consortium inoculated plants also had 66.4% higher capsule weight than that of endophyte-free control plants. More importantly, the consortium of endophytes could enhance the yield of seeds, another economic part of the plant, by 78.8% over control (Table 1). Individual inoculation (SM1B) could only increase the biomass by 31.7%, capsule weight by 30.3% and seed yield by 26.9% which was considerably lower than the consortium inoculated plants. However, SM3B inoculation could only increase the biomass (13.3%) (Table 1).

### Effect of a Consortium of Endophytes on Alkaloid Content

**FIGURE 2:**
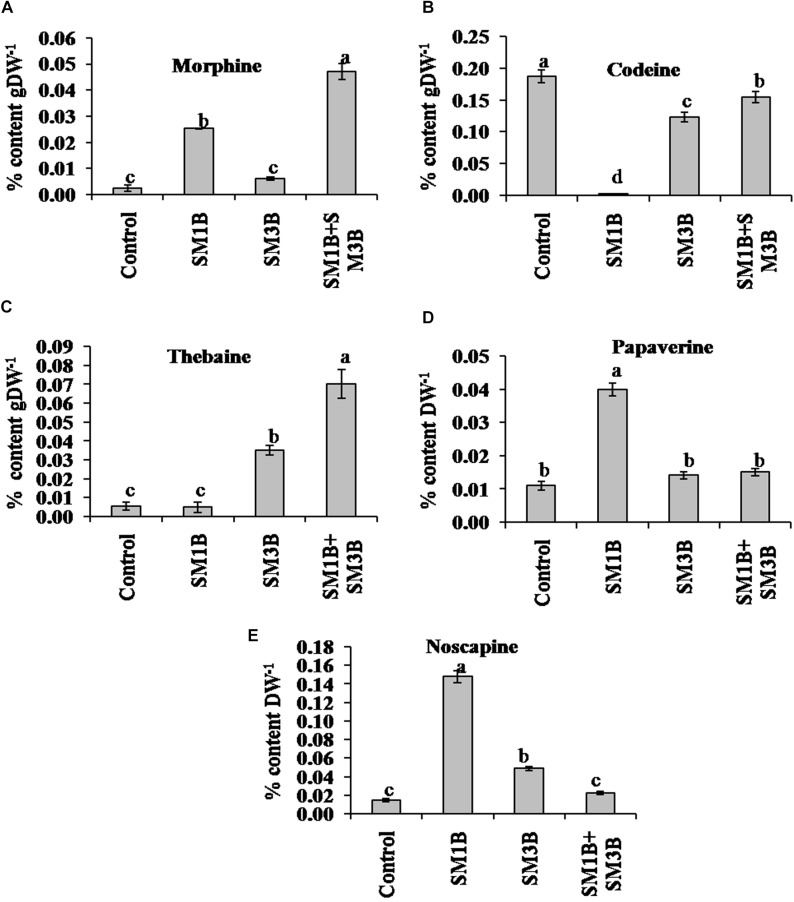
Effect of endophytes inoculation on the alkaloid content of *Papaver somniferum*. Morphine **(A)**, codeine **(B)**, thebaine **(C)**, papaverine **(D),** and noscapine **(E)** content was measured from the dried capsule of 120 days old *P. somniferum* plants inoculated with endophytes SM1B and SM3B individually, and in the form of consortium (SM1B+SM3B). Non-inoculated endophyte free plants were used as a control. The data was an average of two independent quantifications repeated in three biological replicates (*n* = 6) and represented in % content/gram of dry weight (DW). Error bars represent standard errors. Different letters (a–d) indicate statistically significant differences between treatments (Duncan’s multiple range test *P* < 0.05).

To study the impact of a consortium of endophytes on BIA production, the content of morphine, codeine, thebaine, papaverine, and noscapine was estimated in the capsule of individual, consortium inoculated and non-inoculated endophyte-free control poppy plants (Figure 2). Consortium inoculation increased the content of morphine content (2250%) than that of endophyte-free control plants. Consortium inoculation also increased papaverine and noscapine content by 36.4 and 53.3%, respectively.

Individual inoculation of SM1B and SM3B could increase the morphine (200–1150%) which was considerably lower than consortium inoculated plants; also an increase in the content of papaverine (27.3–263.6%) and noscapine (226.7–886.7%) was noticed over non-inoculated plants. Although consortium inoculated plants, had 1067% higher thebaine than that of endophyte-free control plants, inoculation of SM3B could enhance thebaine content only by 483% whereas inoculation SM1B did not affect the thebaine content. Individual inoculation of both the endophytes as well as consortium, on the other hand, decreased the content of codeine (17.6–98.9%).

**FIGURE 3:**
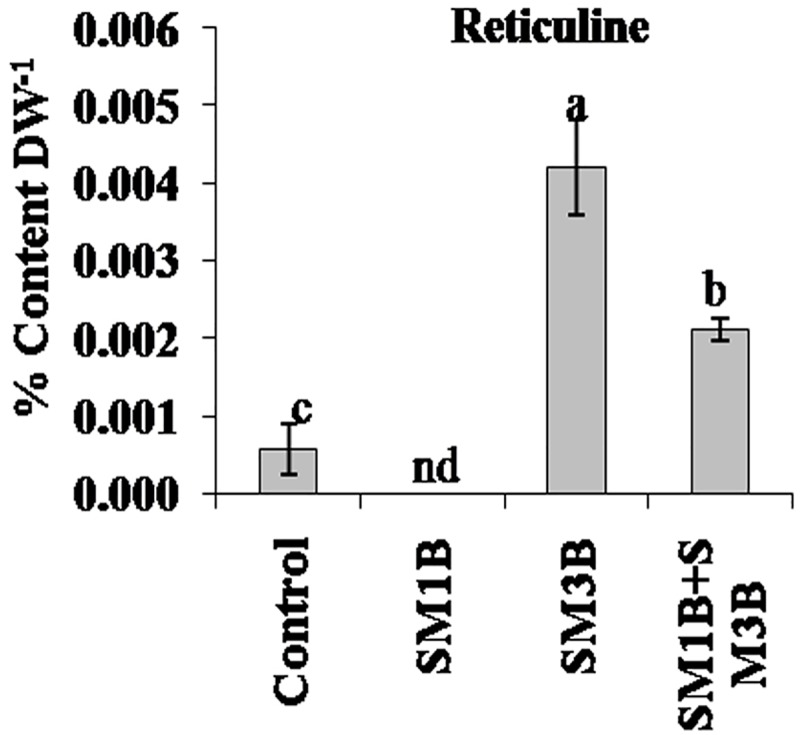
Effect of endophytes inoculation on the reticuline content of *Papaver somniferum*. Reticuline was measured from the dried capsule of 120 days old *P. somniferum* plants inoculated with endophytes SM1B and SM3B individually, and in the form of consortium (SM1B+SM3B). Non-inoculated endophyte free plants were used as a control. The data was an average of two independent quantifications repeated in three biological replicates (*n* = 6) and represented in % content/gram of dry weight (DW). Error bars represent standard errors. Different letters (a–c) indicate statistically significant differences between treatments (Duncan’s multiple range test *P* < 0.05). nd, not detected.

Reticuline content was also found to be affected with the endophyte inoculations. Reticuline was not detected in SM1B-inoculated plants. However, SM3B-inoculated plants had a higher accumulation of reticuline compared to endophyte-free control plants (Figure 3). Consortium-inoculated plants had lower reticuline accumulation than that of SM3B, however, content was higher than that of endophyte-free control plants (Supplementary Table S2).

### Expression of Genes of BIA Biosynthesis

**FIGURE 4:**
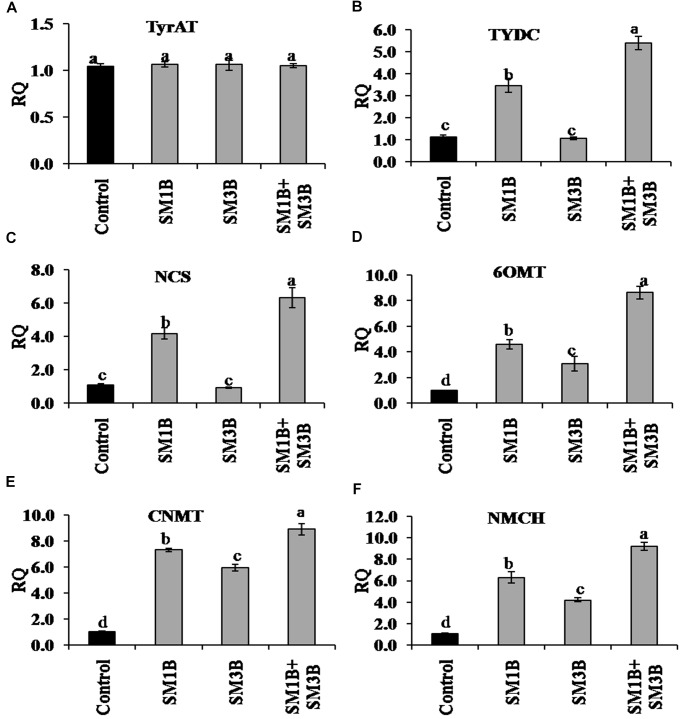
Effect of endophytes inoculation on the expression of genes involved in reticuline biosynthesis. Total RNA was isolated from the green capsule of 90 days old *P. somniferum* plants inoculated with endophytes SM1B and SM3B individually, and in the form of consortium (SM1B+SM3B), reverse transcribed and used as a template for RT-qPCR with SYBR Green detection. The capsules of non-inoculated endophyte free plants were used as a control. Expression of *TyrAT* **(A)**, *TYDC* **(B)**, *NCS* **(C)**, *6OMT* **(D)**, *CNMT* **(E)**, *NMCH* **(F)** was analyzed. Results were normalized to actin (reference transcript) and are shown relative to the level in non-inoculated endophyte free control plants (calibrator). qRT-PCR was performed on triplicate technical replicates of triplicate biological samples (*n = 3*). Data are means ± SE (*n* = 3 biological replicates) and *Y*-axis represents relative quantity (RQ). RQ was calculated using the equation; RQ = 2^−ΔΔCt^. Different letters (a–d) indicate statistically significant differences between treatments (Duncan’s multiple range test *P* < 0.05).

To understand the mechanism of endophytes consortium-mediated increase in morphine content the expression of different genes of BIA biosynthesis was quantified using qRT-PCR. Transcript of a total of 16 genes was quantified. Results were normalized to endogenous gene of opium poppy reference transcript (β-actin) and are presented relative to the level in non-inoculated endophyte-free control plants (calibrator). RQ was calculated using the equation; RQ = 2^−ΔΔCt^. Expression of *TyrAT* was not affected significantly due to the individual as well as consortium inoculations. The expression of *TYDC, NCS*, and *6OMT* in the consortium-inoculated plants was 4.4-, 5.3-, and 7.6-fold higher compared to non-inoculated endophyte-free control plants, respectively (Figure 4). Contrary, SM1B alone inoculated plants had only 2.5- and 3.2-fold higher expression of *TYDC* and *NCS*, respectively, while SM3B inoculation did not affect their expression significantly. *6OMT* expression was lower in individual inoculated plants compared to consortium inoculated plants. Expression of *CNMT* and *NMCH* in consortium-inoculated plants was 8.9- and 9.2-fold higher than that of endophyte-free control plants, respectively (Figure 4). Individual inoculation of SM1B and SM3B could also increase the expression of *CNMT* (5.9–7.3-fold) and *NMCH* (4.2–6.3-fold), but the increment was lower compared to consortium inoculation.

**FIGURE 5:**
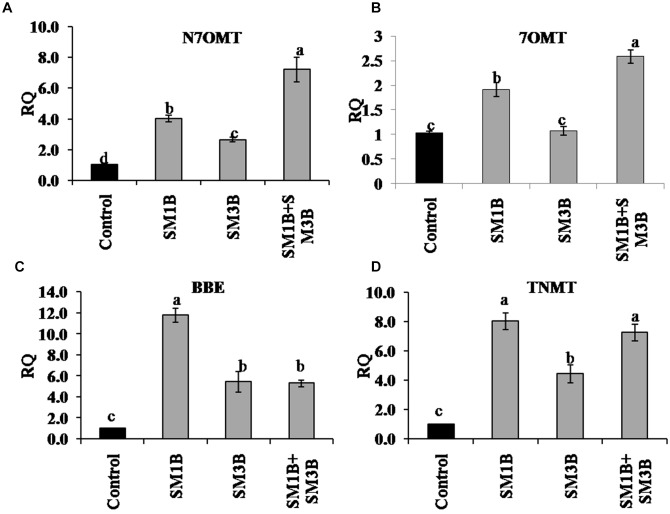
Effect of endophytes inoculation on the expression of genes involved in papaverine and noscapine biosynthesis. Total RNA was isolated from the green capsule of 90 days old *P. somniferum* plants inoculated with endophytes SM1B and SM3B individually, and in the form of consortium (SM1B+SM3B), reverse transcribed and used as a template for RT-qPCR with SYBR Green detection. The capsules of non-inoculated endophyte free plants were used as a control. Expression of genes involved in papaverine biosynthesis *N7OMT* **(A)**, *7OMT* **(B)** and noscapine biosynthesis *BBE* **(C)**, *TNMT* **(D)** was analyzed. Results were normalized to actin (reference transcript) and are shown relative to the level in non-inoculated endophyte free control plants (calibrator). qRT-PCR was performed on triplicate technical replicates of triplicate biological samples (*n = 3*). Data are means ± SE (*n* = 3 biological replicates) and *Y*-axis represents relative quantity (RQ). RQ was calculated using the equation; RQ = 2^−ΔΔCt^. Different letters (a–d) indicate statistically significant differences between treatments (Duncan’s multiple range test *P* < 0.05).

Expression of genes involved in papaverine biosynthesis, i.e., *N7OMT* and *7OMT* was increased by 7.2- and 2.6-fold, in consortium-inoculated plants, respectively (Figure 5). Individual inoculation could also upregulate *N7OMT* and *7OMT* expression but were lower than that of consortium inoculated plants. In consortium-inoculated plants expression of genes involved in noscapine biosynthesis, i.e., *BBE* and *TNMT* was increased by 5.3- and 7.3-fold, respectively (Figure 5) whereas individual-inoculation could also increase *BBE* (5.5–11.8-fold) and *TNMT* (4.5–8-fold) expression.

**FIGURE 6:**
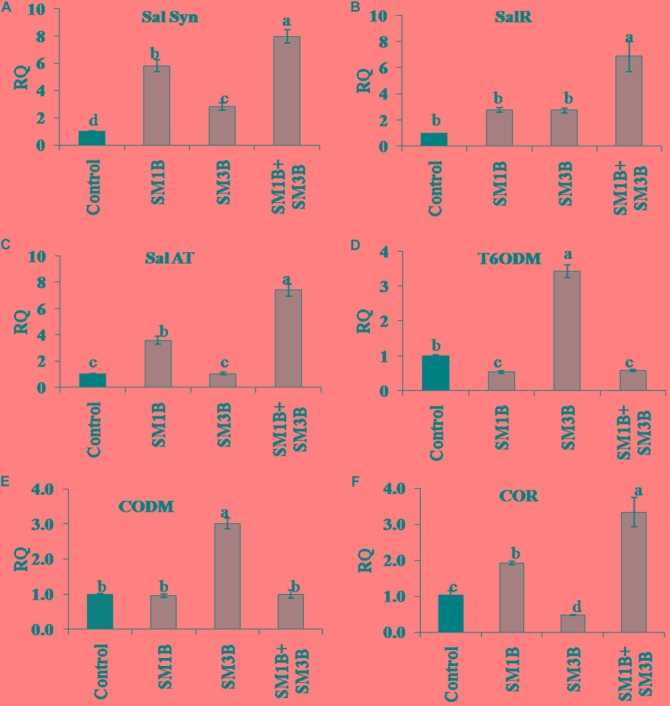
Effect of endophytes inoculation on the expression of genes involved in morphine biosynthesis. Total RNA was isolated from the green capsule of 90 days old *P. somniferum* plants inoculated with endophytes SM1B and SM3B individually, and in the form of consortium (SM1B+SM3B), reverse transcribed and used as a template for RT-qPCR with SYBR Green detection. The capsules of non-inoculated endophyte free plants were used as a control. Expression of *SalSyn* **(A)**, *SalR* **(B)**, *SalAT* **(C)**, *T6ODM* **(D)**, *CODM* **(E)**, *COR* **(F)** was analyzed. Results were normalized to actin (reference transcript) and are shown relative to the level in non-inoculated endophyte free control plants (calibrator). qRT-PCR was performed on triplicate technical replicates of triplicate biological samples (*n = 3*). Data are means ± SE (*n* = 3 biological replicates) and *Y*-axis represents relative quantity (RQ). RQ was calculated using the equation; RQ = 2^−ΔΔCt^. Different letters (a–d) indicate statistically significant differences between treatments (Duncan’s multiple range test *P* < 0.05).

**FIGURE 7:**
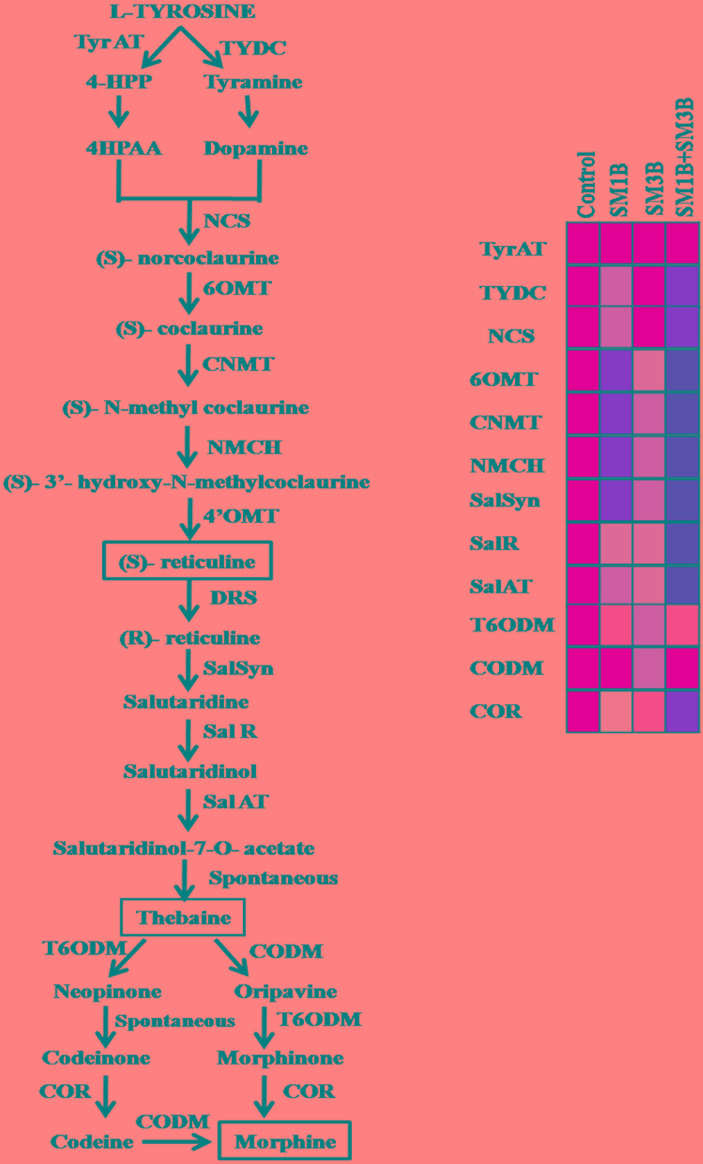
Upregulation of multiple genes involved in morphine biosynthesis due to inoculation of a consortium of endophytes. SM1B (*Acinetobacter*) endophytes upregulated the expression of most of the genes of BIAs pathway except *T6ODM* and *CODM* whereas SM3B (*Marmoricola* sp.) inoculation upregulated the *T6ODM* and *CODM*. Consortium-inoculation (SM1B+SM3B) could enhance the morphine production by upregulating, the expression of most of the BIA biosynthetic genes (*TYDC*, *NCS*, *6OMT*, *CNMT*, *NMCH*, *SalSyn*, *SalR*, *SalAT*, *COR*) involved in morphine biosynthesis to a greater extent, than that of single-inoculations. Expression of different genes was presented in square boxes. Red color of boxes indicate upregulated expression (intensity of red color shows the level of expression, i.e., more red more expression and vice versa), yellow color shows the level of expression in non-inoculated endophyte free control plants and light yellow color shows downregulated expression. *Enzyme abbreviations:* TyrAT, tyrosine aminotransferase; TYDC, tyrosine/ dopa decarboxylase; NCS, norcoclaurine synthase; 6OMT, (S)-norcoclaurine 6-O-methyltransferase; CNMT, (S)- coclaurine N-methyltransferase; NMCH, (S)-N-methylcoclaurine 3-hydroxylase; 4-OMT, (S)-30-hydroxy-N-methylcoclaurine 4-O-methyltransferase; DRS, 1,2-dehydroreticuline synthase; SalSyn, salutaridine synthase; SalR, salutaridine reductase; SalAT, salutaridinol 7-O-acetyltransferase; T6ODM, thebaine 6-O-demethylase; COR, codeinone reductase; CODM, codeine O-demethylase.

The increment in the expression of *SalSyn*, *SalR*, and *SalAT* mediated by consortium inoculation was higher than the individual endophyte inoculation (Figure 6). Consortium-inoculated plants had 7.0-, 5.9-, and 6.4-fold higher *SalSyn*, *SalR*, and *SalAT* expression compared to non-inoculated endophyte-free control plants. Individual inoculation of SM1B and SM3B could increase the expression of *SalSyn* (2.8–5.8-fold), *SalR* (2.7–2.8-fold) and *SalAT* (3.6-fold) to lower levels only. The consortium-inoculated plants and SM1B inoculated plants had reduced expression of *T6ODM*, however, SM3B inoculated plants had 2.4-fold higher *T6ODM* expression compare to endophyte-free control plants (Figure 6). Expression of *CODM* was not affected significantly due to the consortium- and SM1B-inoculation. However, SM3B inoculated plants had almost twofold higher *CODM* expression than the endophyte-free control plants, SM1B and consortium inoculated plants (Figure 6). Expression of *COR*, considered to be the most critical regulatory gene, was 2.3-fold higher in the consortium- inoculated plants whereas SM1B alone could increase it to the level of 0.9-folds only and SM3B inoculation reduced *COR* expression compare to endophyte-free control (Figure 6). Upregulation of multiple genes involved in morphine biosynthesis due to inoculation of a consortium of endophytes is represented in Figure 7 (Supplementary Table S3).

## Discussion

Due to limitations related to social acceptability and cost-effectiveness in genetic manipulation approaches such as the development of transgenics by overexpressing single or multiple genes simultaneously, there is a need of an alternate sustainable approach for improvement of crop production and a healthier environment. Microbes associated with plants such as plant growth promoting rhizobacteria (PGPRs) and endophytes have been used as promising candidates for sustainable agriculture. They are found to be involved in improving plant growth and protection from environmental stresses. We have demonstrated that PGPRs and endophytes can protect the plant from environmental stresses by modulating the phytohormone status of plants and by modulating the expression of stress-responsive genes (Bharti et al., 2016; Barnawal et al., 2016, 2017). Also, endophytes residing in most of the parts of the plant including root, stem, leaves, seeds, fruits, flower, and found to perform the tissue-specific function may also modulate primary and secondary metabolism of a plant (Pandey et al., 2016a). Endophytes are considered better candidates than PGPR because they function residing inside the plant and therefore, they do not face any competition with the resident soil microorganisms and also escape from the surrounding harsh environmental conditions. Diverse endophytes are available with specific characteristics related to growth promotion ability and stress tolerance such as an ability of nutrient acquisition, nitrogen fixation, phosphate solubilization, ACC-deaminase production, phytohormone production and suppression of plant pathogens that make them useful for improvement of plant growth and for providing tolerance to environmental stresses. A single endophyte may not be able to carry said multiple growth promotion activities. Therefore, to obtain maximum growth and crop yield consortium of endophytes having different characteristics will be a better approach than single-endophyte inoculation.

Previously, we have demonstrated that inoculation of an endophyte of opium poppy SM1B (*Acinetobacter* sp.) which was isolated from the capsule of an alkaloid-rich cultivar of *P. somniferum* (cv. Sampada) enhanced morphine content substantially in alkaloid-less cv. Sujata. However, the accumulation of intermediates of morphine synthesis; thebaine and codeine was very low in SM1B-inoculated plants (Pandey et al., 2016b). Also, another endophyte SM3B (*Marmoricola* sp.) inoculation could not increase the morphine content significantly, but substantially increased the thebaine (another important alkaloid) accumulation compared to endophyte-free control plants. Gene expression study of these endophytes (SM1B and SM3B) inoculated plants showed that most of the genes (except *T6ODM* and *CODM*) of BIA biosynthesis were upregulated in SM1B-inoculated plants. On the other hand, *T6ODM* and *CODM* could be upregulated by SM3B-inoculation. The present study therefore aimed at upregulation of all the genes involved in morphine biosynthesis by inoculating these two endophytes through complementarity. Single-inoculation of these endophytes enhanced the morphine content in a morphine-less cultivar of *P. somniferum* (cv. Sujata), however, this could not increase up to the level of an alkaloid-rich cultivar of *P. somniferum* (cv. Sampada) from where these endophytes were isolated. Therefore, the efficacy of combined inoculation of both the endophytes (SM1B and SM3B) in maximizing the production of morphine, was tested in *P. somniferum* cv. Sujata which is otherwise morphine-less variety.

Both the endophytes (SM1B and SM3B) were able to survive and perpetuate in combination and could colonize successfully in the capsule of the poppy plant when applied as a consortium. However, consortium inoculation slightly reduced the colonization of SM1B compared to individual SM1B inoculation. Consortium inoculation could enhance the growth of *P. somniferum* cv. Sujata plants compared to individual inoculations by increasing the content of photosynthetic pigments, photosynthetic rate, transpiration rate and stomatal conductance. The increased photosynthetic efficiency of the consortium-inoculated poppy plant could enhance the biomass (73.3%), capsule weight (66%), and seed yield (79%) which was substantially higher than single-inoculation.

Combined-inoculation of SM1B and SM3B could generate higher increments in morphine content compared to individual-inoculations by modulating the utilization of the key intermediates (reticuline and thebaine) of morphine biosynthesis. Enhanced morphine content was due to more thebaine production in consortium inoculated plants (0.07% content gDW^−1^) compared to individual-inoculation (0.005% content gDW^−1^ in case of SM1B and 0.035% content gDW^−1^ in case of SM3B). Higher thebaine production could result in lower accumulation of reticuline (upstream intermediate) in consortium inoculated plants compare to single inoculation of SM3B. However, very low accumulation of reticuline and thebaine indicates complete utilization of these intermediates for morphine biosynthesis in the case of SM1B-inoculated plants.

Consortium-inoculation could enhance the morphine production by upregulating, the expression of most of the BIA biosynthetic genes (*TYDC*, *NCS*, *6OMT*, *CNMT*, *NMCH*, *SalSyn*, *SalR*, *SalAT*, *COR*) involved in morphine biosynthesis to a greater extent, than that of single-inoculations (Figure 7). Expression of *TYDC*, *NCS*, *6OMT*, *CNMT*, *NMCH* which are the genes upstream to reticuline biosynthesis was higher in consortium inoculated plants, clearly indicating greater flux of BIA biosynthesis toward central intermediate, i.e., reticuline. However, due to the increased expression of *SalSyn*, *SalR*, and *SalAT* (which was higher in consortium-inoculated plants than that of single-inoculation); reticuline accumulation was found to be low than that of SM3B-inoculated plants. Due to higher expression of *SalSyn*, *SalR*, and *SalAT* than that of single inoculation; conversion of reticuline to thebaine was higher, and that could have resulted in more thebaine accumulation in consortium-inoculated plants compared to single-inoculated plants.

Here, we probably for the first time demonstrated that endophytes affecting the host metabolism at gene expression level having different modes/targets of action could be combined to compliment the inability of one endophyte to upregulate certain genes thus improving the biosynthesis of secondary metabolites. Also, by using the consortium of different endophytes with diverse functional growth attributes, we can compliment the functional deficiency of one by another. These endophytes would be a better alternative in place of transgenic plants and also could be explored for the development of designer plants. Here, in the present study, we also established that consortium of selected endophytes could be applied to overexpress multiple genes to increase the plant yield/desired product. The present study was conducted in morphine-less genotype Sujata to establish the role of combined inoculation of endophytes in enhancing morphine, and therefore the magnitude of enhancement was quite high considering very low concentrations inherently present in genotype Sujata. These enhancements, however, might not be possible in morphine/alkaloid rich variety(ies). It is, however, essential to understand the detailed mechanism of action of promising endophytes, and then a combined approach can be applied to attain maximum yields.

## Author Contributions

AK, TR, and SP conceived and designed the experiments and wrote the manuscript. TR and MS performed the experiments. AK, KS, TR, SP, and AP analyzed the data. All authors read and approved the manuscript.

## Conflict of Interest Statement

The authors declare that the research was conducted in the absence of any commercial or financial relationships that could be construed as a potential conflict of interest.
