# Supplementary material for: Endophytic Consortium With Diverse Gene-Regulating Capabilities of Benzylisoquinoline Alkaloids Biosynthetic Pathway Can Enhance Endogenous Morphine Biosynthesis in Papaver somniferum
Source: Front Microbiol. 2019 Apr 30;10:925. doi: 10.3389/fmicb.2019.00925 (PMC6503101; doi:10.3389/fmicb.2019.00925)
Supplement: Supplementary file 1 [file Data_Sheet_1.docx]

**Endophytic consortium with diverse gene-regulating capabilities of benzylisoquinoline alkaloids biosynthetic pathway can enhance endogenous morphine biosynthesis in *Papaver somniferum***

**Supplementary Figure S1** Antagonistic activity of endophytes
