# Supplementary material for: Endophytic Consortium With Diverse Gene-Regulating Capabilities of Benzylisoquinoline Alkaloids Biosynthetic Pathway Can Enhance Endogenous Morphine Biosynthesis in Papaver somniferum
Source: Front Microbiol. 2019 Apr 30;10:925. doi: 10.3389/fmicb.2019.00925 (PMC6503101; doi:10.3389/fmicb.2019.00925)
Supplement: Supplementary file 2 [file Data_Sheet_2.docx]

**Endophytic consortium with diverse gene-regulating capabilities of benzylisoquinoline alkaloids biosynthetic pathway can enhance endogenous morphine biosynthesis in *Papaver somniferum***

**Supplementary Table S1.** Colonization of endophytes in the capsule of *Papaver somniferum* plants. CFU- colony forming unit. Values are the mean ± SE of three replicates (*n* =3). Different letters (a-b) indicate statistically significant differences between treatments (Duncan’s multiple range test *P*<0.05).

| Number of CFU within plant tissue  (log_10_ CFU/gFW tissue) | SM1B | SM3B | Consortium | |
| --- | --- | --- | --- | --- |
|  |  |  | SM1B | SM3B |
| Mean±SE | 4.00±0.18^a^ | 3.3±0.29^b^ | 3.56±0.18^b^ | 3.36±0.19^b^ |

**Supplementary Table S2.** Effect of endophytes inoculation on the alkaloid content of *Papaver somniferum*. Morphine, codeine, thebaine, papaverine and noscapine and reticuline content was measured from the dried capsule of 120-days old *P. somniferum* plants inoculated with endophytes SM1B and SM3B individually, and in the form of consortium (SM1B+SM3B). Non-inoculated endophyte free plants were used as a control. The data was an average of two independent quantifications repeated in three biological replicates (*n* =6) and represented in % content/gram of dry weight (DW). Error bars represent standard errors. Different letters (a-d) indicate statistically significant differences between treatments (Duncan’s multiple range test *P*<0.05).

| BIAs (% Content DW^-1^) | Control | SM1B | SM3B | Consortium |
| --- | --- | --- | --- | --- |
|  | Mean±SE | Mean±SE | Mean±SE | Mean±SE |
| Morphine | 0.002±0.001^c^ | 0.025±0.0002^b^ | 0.006±0.0006^c^ | 0.047±0.003^a^ |
| Codeine | 0.187±0.010^a^ | 0.002±0.0008^d^ | 0.124±0.008^c^ | 0.154±0.009^b^ |
| Thebaine | 0.006±0.002^c^ | 0.005±0.003^c^ | 0.035±0.002^b^ | 0.070±0.008^a^ |
| Papaverine | 0.011±0.001^b^ | 0.040±0.002^a^ | 0.014±0.001^b^ | 0.015±0.001^b^ |
| Noscapine | 0.015±0.002^c^ | 0.148±0.006^a^ | 0.049±0.002^b^ | 0.023±0.002^c^ |
| Reticuline | 0.0006±0.0003^c^ | 0 (nd) | 0.0042±0.0006^a^ | 0.0021±0.0001^b^ |

**Supplementary Table S3.** Effect of endophytes inoculation on the expression of genes involved in BIA biosynthesis. Total RNA was isolated from the green capsule of 90-days old *P. somniferum* plants inoculated with endophytes SM1B and SM3B individually, and in the form of consortium (SM1B+SM3B), reverse transcribed and used as a template for RT-qPCR with SYBR Green detection. The capsules of non-inoculated endophyte free plants were used as a control. Expression of *TyrAT*, *TYDC*, *NCS*, *6OMT*, *CNMT*, *NMCH*, *N7OMT*, *7OMT,* *BBE, TNMT, SalSyn*, *SalR*, *SalAT,* *T6ODM*, *CODM* and *COR* was analyzed. Results were normalized to actin (reference transcript) and are shown relative to the level in non-inoculated endophyte free control plants (calibrator). qRT-PCR was performed on triplicate technical replicates of triplicate biological samples (*n=3*). Data are means ± SE (*n* = 3 biological replicates) and *Y*-axis represents relative quantity (RQ). RQ was calculated using the equation; RQ= 2^-∆∆Ct^. Different letters (a-d) indicate statistically significant differences between treatments (Duncan’s multiple range test *P*<0.05).

| Gene | Control | SM1B | SM3B | Consortium |
| --- | --- | --- | --- | --- |
|  | RQ ±SE | RQ ±SE | RQ±SE | RQ ±SE |
| TyrAT | 1.04±0.02^a^ | 1.06±0.03^a^ | 1.06±0.06^a^ | 1.05±0.02^a^ |
| TYDC | 1.11±0.09^c^ | 3.46±0.31^b^ | 1.05±0.06^c^ | 5.39±0.30^a^ |
| NCS | 1.08±0.07^c^ | 4.17±0.33^b^ | 0.93±0.02^c^ | 6.31±0.60^a^ |
| 6OMT | 1.02±0.01^d^ | 4.60±0.36^b^ | 3.09±0.58^c^ | 8.64±0.49^a^ |
| CNMT | 1.03±0.02^d^ | 7.32±0.14^b^ | 5.96±0.27^c^ | 8.92±0.42^a^ |
| NMCH | 1.07±0.06^d^ | 6.29±0.53^b^ | 4.19±0.19^c^ | 9.20±0.36^a^ |
| N7OMT | 1.03±0.08^d^ | 4.03±0.23^b^ | 2.65±0.15^c^ | 7.22±0.79^a^ |
| 7OMT | 1.04±0.03^c^ | 1.91±0.14^b^ | 1.41±0.09^c^ | 2.59±0.13^a^ |
| BBE | 1.03±0.01^c^ | 11.75±0.66^a^ | 5.45±1.00^b^ | 5.30±0.31^b^ |
| TNMT | 1.02±0.01^c^ | 8.05±0.58^a^ | 4.45±0.60^b^ | 7.27±0.59^a^ |
| SalSyn | 1.02±0.01^d^ | 5.81±0.44^b^ | 2.82±0.26^c^ | 7.96±0.49^a^ |
| SalR | 1.01±0.01^b^ | 2.77±0.17^b^ | 2.73±0.19^b^ | 6.88±1.15^a^ |
| SalAT | 1.04±0.03^c^ | 3.58±0.30^b^ | 1.04±0.10^c^ | 7.42±0.46^a^ |
| T6ODM | 1.01±0.03^b^ | 0.54±0.04^c^ | 3.43±0.19^a^ | 0.58±0.03^c^ |
| CODM | 0.99±0.03^b^ | 0.95±0.04^b^ | 3.02±0.15^a^ | 0.99±0.11^b^ |
| COR | 1.04±0.05^c^ | 1.92±0.05^b^ | 0.47±0.01^d^ | 3.34±0.08^a^ |
